# Complete mitochondrial genome of white-striped long-horned beetle, *Batocera lineolata* (Coleoptera: Cerambycidae) by next-generation sequencing and its phylogenetic relationship within superfamily Chrysomeloidea

**DOI:** 10.1080/23802359.2017.1361797

**Published:** 2017-08-09

**Authors:** Jian-Hong Liu, Ping-Fan Jia, Tong Luo, Qing-Mei Wang

**Affiliations:** Key Laboratory of Forest Disaster Warning and Control in Yunnan Province, Southwest Forestry University, Kunming, China

**Keywords:** *Batocera lineolata*, mitochondrial genome, molecular phylogeny

## Abstract

White-striped long-horned beetle, *Batocera lineolata* occurred in China, Vietnam, Myanmar, India, Japan and Korea and is one of the most important wood-boring forestry pests. Complete mitochondrial genome was determined based on next-generation sequencing. The long-horned beetle has a total length of 15,420 bp, consisting of 13 protein-coding genes (PCGs), 22 tRNA, 2 rRNA genes and a non-coding region. The phylogenetic position was closely clustered with 6 other Lamiinae species with strong node support. Within Chrysomeloidea superfamily, a monophyletic Lamiinae clad is recovered as the sister group of the Cerambycinae from three species and the Chrysomelidae consisting five species forms a monophyletic clade which is strongly supported.

Chrysomeloidea are an enormous superfamily of Coleoptera beetles, mostly in the families Chrysomelidae, the leaf beetles and Cerambycidae, the long horned beetles. Many species in these two families are important plant pests. White-striped long horned beetle, *Batocera lineolata* (Coleoptera: Cerambycidae), mainly distributed in China, Vietnam, Myanmar, India, Japan and Korea (Yang et al. 2013). The long-horned beetle is one of the most important wood-boring forestry pests and can attack more than 20 host species that are taxonomically distant plant families (Gao et al. 1995; Liang et al. 2008; Li et al. 2008, 2009; Yang et al. 2013). The larvae of *B. lineolata*, called round-headed borers can cause extensive damage to living trees by boring into trunk. This species occurs one generation every 2 years and overwinters as larvae or adults in tree trunks (Yu et al. 2007). *Viburnum awabuki* (Dipsacales: Adoxaceae), is considered to be the preferred host plant for adults (Liang et al. 2008; Yang et al. 2011).

In present study, the complete mitogenome of *B. lineolata* was determined based on next-generation sequencing. Molecular phylogeny was analysed using complete mitogenome from 14 Chrysomeloidea beetles. Samples were collected from Daguanlou Park (25.03°N, 102.67°E), located in Kunming, Capital of Yunnan Province of China, in May, 2015. Specimen was deposited in Yunnan Provincial Key Laboratory of Forest Disaster Warning and Control, Kunming, China with a voucher number KM20150516. Genomic DNA was extracted following the manufacturer’s instruction in the DNeasy Blood and Tissue kit (Qiagen) for animal tissue (spin column). Whole genome shotgun reads were sequenced with the Illumina sequencing platform and complete mitochondrial genome of *B. lineolata* were assembled with high coverage using Illumina sequencing data. The complete mitogenome of *B. lineolata* sequenced in the present study is circular double-strand DNA molecule. The genome sizes are 15,420 bp of length which is not within the range reported in other completely sequenced Chrysomeloidea superfamily, ranging from 15,505 bp in *Thyestilla gebleri* to 16,650 bp in *Diabrotica virgifera virgifera*. The mitogenome contains 13 protein-coding genes (NAD1-6, NAD4l, COX1-3, CYTB, ATP6 and ATP8), 22 tRNA, 2 rRNA genes and a non-coding region (A + T-rich control region). J-strand (plus strand) codes 9 PCGs and 14 tRNAs, and the other genes, 4 PCGs, 8 tRNAs and 2 rRNAs, are coded in N-strand (minus strand). Gene arrangement is identical to the most common type of the putative ancestor of insects (Boore, 1999; Cameron, 2014). The nucleotide composition of *B. lineolata* mitochondrial genome is biased toward adenine and thymine (accounting for 74.5%: A = 38.8%, T = 35.7%, G = 9.3% and C = 16.2%). This bias is well within the range detected for the sequenced Chrysomeloidea species, from 69.5% in *Spiniphilus spinicornis* to 79.8% in *Paleosepharia posticata*. Maximum likelihood phylogenetic analysis was performed using mitochondrial genome data sets (Figure 1). The phylogenetic position of *B. lineolata* was closely clustered with 6 other Lamiinae species with strong node support. Within Chrysomeloidea superfamily, the Chrysomelidae, including *Agasicles hygrophila, Galeruca daurica, Paleosepharia posticata, Diabrotica barberi* and *Diabrotica virgifera virgifera*, forms a monophyletic clade which is strongly supported. A monophyletic Lamiinae clad (*B. lineolate* and the other six species) is recovered as the sister group of the Cerambycinae, consisting of *Spiniphilus spinicornis, Aeolesthes oenochrous and Massicus raddei*.

**Figure 1.**
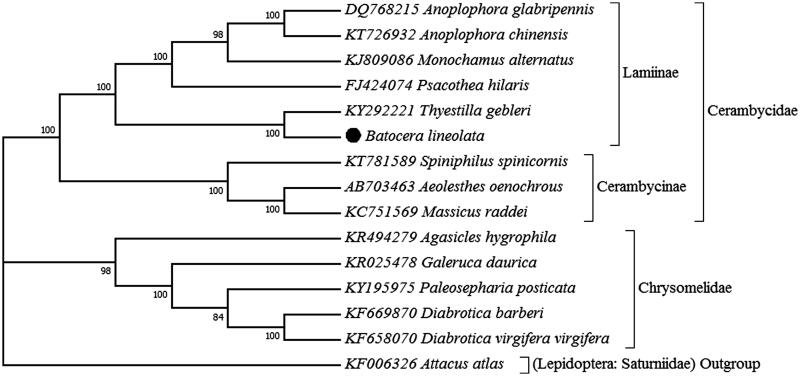
Molecular phylogenetic analysis within superfamily Chrysomeloidea by Maximum Likelihood method. Genbank accessions were indicated with species scientific name. The phylogenetic position was marked in solid circle shape for white-striped long-horned beetle.
